# 5-(2,5-Dioxooxolan-3-yl)-8-methyl-3,3a,4,5-tetra­hydro-1*H*-naphtho­[1,2-*c*]furan-1,3-dione

**DOI:** 10.1107/S1600536813000482

**Published:** 2013-01-12

**Authors:** Y. Z. Guo, J. G. Liu, S. Y. Yang

**Affiliations:** a Laboratory of Advanced Polymer Materials, Institute of Chemistry, Chinese Academy of Sciences (ICCAS), Beijing 100190, People’s Republic of China

## Abstract

In the title compound, C_17_H_14_O_6_, the dihedral angle between the two anhydride rings is 76.01 (8)°while the dihedral angles between the benzene and anhydride rings are 42.60 (7) and 68.94 (7)°. The cyclo­hexene ring of the tetra­hydro­naphthalene unit exhibits an envelope conformation.

## Related literature

For applications of tetra­lin-containing dianhydrides, see: Liaw *et al.* (2012 ▶); Matsumoto *et al.* (2009 ▶); Hasegawa & Horie (2001 ▶). For puckering parameters, see: Cremer & Pople (1975 ▶).
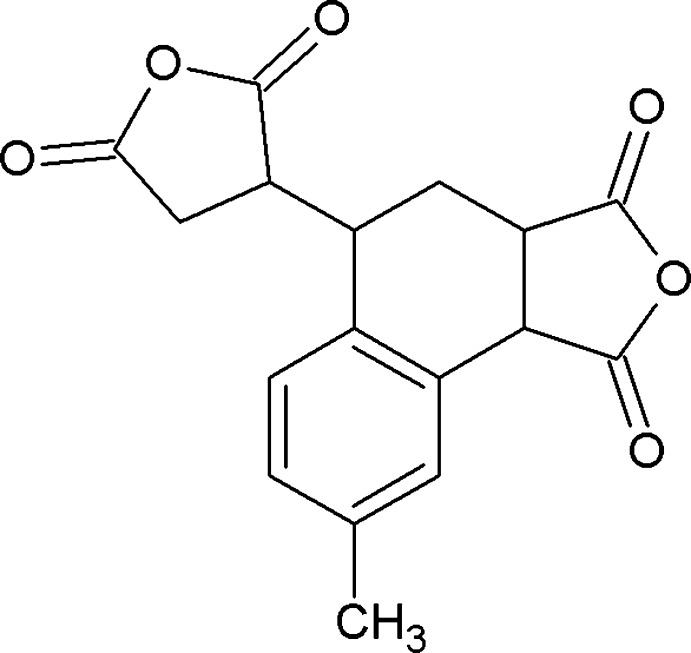


## Experimental

### 

#### Crystal data

C_17_H_14_O_6_
*M* *_r_* = 314.28
Triclinic, 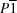

*a* = 6.6907 (13) Å
*b* = 9.166 (2) Å
*c* = 11.839 (2) Å
α = 78.628 (8)°
β = 78.352 (9)°
γ = 79.054 (9)°
*V* = 688.5 (2) Å^3^
*Z* = 2
Mo *K*α radiation
μ = 0.12 mm^−1^
*T* = 173 K
0.28 × 0.22 × 0.12 mm

#### Data collection

Rigaku Saturn724+ CCD diffractometer
Absorption correction: multi-scan (*CrystalClear*; Rigaku, 2008 ▶) *T*_min_ = 0.680, *T*_max_ = 1.000
8959 measured reflections
3137 independent reflections
2864 reflections with *I* > 2σ(*I*)
*R*_int_ = 0.042

#### Refinement

*R*[*F* ^2^ > 2σ(*F* ^2^)] = 0.046
*wR*(*F* ^2^) = 0.113
*S* = 1.07
3137 reflections
209 parameters
H-atom parameters constrained
Δρ_max_ = 0.32 e Å^−3^
Δρ_min_ = −0.22 e Å^−3^

### 

Data collection: *CrystalClear* (Rigaku, 2008 ▶); cell refinement: *CrystalClear*; data reduction: *CrystalClear*; program(s) used to solve structure: *SHELXS97* (Sheldrick, 2008 ▶); program(s) used to refine structure: *OLEX2* (Dolomanov *et al.*, 2009 ▶); molecular graphics: *OLEX2*; software used to prepare material for publication: *SHELXL97* (Sheldrick, 2008 ▶).

## Supplementary Material

Crystal structure: contains datablock(s) I, global. DOI: 10.1107/S1600536813000482/vm2186sup1.cif

Supplementary material file. DOI: 10.1107/S1600536813000482/vm2186Isup2.cdx

Structure factors: contains datablock(s) I. DOI: 10.1107/S1600536813000482/vm2186Isup3.hkl

Supplementary material file. DOI: 10.1107/S1600536813000482/vm2186Isup4.cml

Additional supplementary materials:  crystallographic information; 3D view; checkCIF report

## Figures and Tables

**Fig. 1.**
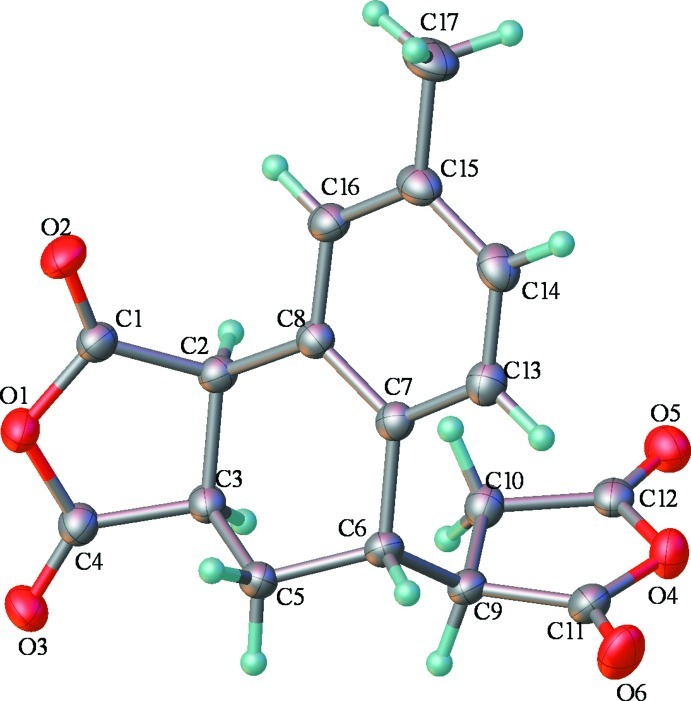
The molecular structure of the title compound showing displacement ellipsoids at the 30% probability level.

## supplementary crystallographic information

### Comment

The title compound 3,4-dicarboxy-1,2,3,4-tetrahydro-6-methyl-1-naphthalene succinic dianhydride (MTDA) can be used as monomer for alicyclic polyimide (PI) synthesis (Liaw *et al.*, 2012). Especially, this dianhydride compound is a very promising monomer for developments of organo-soluble and highly-transparent or colorless PI films. Colorless polyimide films have recently attracted much attention in optoelectronic fabrications, such as plastic substrate for flexible display, waveguides for optical interconnection and so on, due to their excellent combined properties, including high thermal stability, high optical transparency, and low dielectric constant (Matsumoto *et al.*, 2009). The asymmetrical alicyclic tetralin moiety in MTDA effectively reduces the inter- or intramolecular interactions and prohibits the formation of charge transfer complex (Hasegawa *et al.*, 2001); thus improving the optical transparency and solubility of the derived PIs.

The title compound has an asymmetrical structure (Fig. 1). The dihedral angle between the best planes through the two anhydride rings is 76.01 (8)°. The dihedral angles between the benzene ring and the anhydride ring 1 (C1-C4/O1) and anhydride ring 2 (C9-C12/O4) is 42.60 (7)° and 68.94 (7)°, respectively. The six-membered cyclohexene ring in the tetrahydronaphthalene residue exhibits an envelope conformation with puckering parameters of Q=0.489 (15) Å, θ=122.8 (2)° and φ=300.7 (2)° (Cremer *et al.*, 1975).

### Experimental

Maleic anhydride (43.75 g, 0.446 mol), 4-methylstyrene (80.60 g, 0.682 mol), and 2,5-di-*tert*-butyl hydroquinone (0.1138 g, 0.5 mmol) were put into a 500-ml three-necked flask equipped with a mechanical stirrer, gas inlet, and condenser. Nitrogen was first introduced to remove the air in the system. Then, nitric oxide (NO) was introduced from a gas inlet under the surface of the reaction solution. The reaction mixture were heated to 120°C and maintained for 5 h under an atmosphere of nitric oxide. The produced red-brown nitrogen oxide gas was trapped by passing through an aqueous solution of 20 wt% sodium hydroxide. An orange precipitate formed during the reaction. After the reaction, the mixture was cooled to room temperature and 60 ml of acetonitrile was then added. The reaction mixture was heated to reflux for another 0.5 h. Then, 60 ml of toluene was added and the reaction mixture was cooled to room temperature. The produced white needlelike crystals were collected by filtration and the solid was washed in succesion with toluene and petroleum ether. The obtained white solids were dried in vacuum at 80°C for 24 h. Yield: 51.44 g (73.4%). Elemental analysis: calculated for C_17_H_14_O_6_: C 64.97, H 4.49%. Found: C 64.32, H 4.44%. EI—MS, m/*z*: 142 (*M*^+^-172, 100%). Colorless single crystals were grown by slow evaporation of an acetonitrile solution over a period of several days.

### Refinement

H atoms were positioned geometrically (C—H=0.95–1.00 Å) and refined using a riding model with the *U*_iso_(H)=1.2 *U*_eq_(C).

### Crystal data

**Table:**

C_17_H_14_O_6_	*Z* = 2
*M**_r_* = 314.28	*F*(000) = 328
Triclinic, *P*1	*D*_x_ = 1.516 Mg m^−^^3^
Hall symbol: -P 1	Melting point: 512 K
*a* = 6.6907 (13) Å	Mo *K*α radiation, λ = 0.71073 Å
*b* = 9.166 (2) Å	Cell parameters from 2461 reflections
*c* = 11.839 (2) Å	θ = 2.3–27.5°
α = 78.628 (8)°	µ = 0.12 mm^−^^1^
β = 78.352 (9)°	*T* = 173 K
γ = 79.054 (9)°	Plate, colourless
*V* = 688.5 (2) Å^3^	0.28 × 0.22 × 0.12 mm

### Data collection

**Table:**

Rigaku Saturn724+ CCD diffractometer	3137 independent reflections
Radiation source: sealed tube	2864 reflections with *I* > 2σ(*I*)
Graphite monochromator	*R*_int_ = 0.042
ω scans at fixed χ = 45°	θ_max_ = 27.5°, θ_min_ = 3.2°
Absorption correction: multi-scan (*CrystalClear*; Rigaku, 2008)	*h* = −8→8
*T*_min_ = 0.680, *T*_max_ = 1.000	*k* = −11→11
8959 measured reflections	*l* = −15→15

### Refinement

**Table:**

Refinement on *F*^2^	Primary atom site location: structure-invariant direct methods
Least-squares matrix: full	Secondary atom site location: difference Fourier map
*R*[*F*^2^ > 2σ(*F*^2^)] = 0.046	Hydrogen site location: inferred from neighbouring sites
*wR*(*F*^2^) = 0.113	H-atom parameters constrained
*S* = 1.07	*w* = 1/[σ^2^(*F*_o_^2^) + (0.0473*P*)^2^ + 0.2684*P*] where *P* = (*F*_o_^2^ + 2*F*_c_^2^)/3
3137 reflections	(Δ/σ)_max_ < 0.001
209 parameters	Δρ_max_ = 0.32 e Å^−^^3^
0 restraints	Δρ_min_ = −0.22 e Å^−^^3^

### Special details

**Table:**

Geometry. All e.s.d.'s (except the e.s.d. in the dihedral angle between two l.s. planes) are estimated using the full covariance matrix. The cell e.s.d.'s are taken into account individually in the estimation of e.s.d.'s in distances, angles and torsion angles; correlations between e.s.d.'s in cell parameters are only used when they are defined by crystal symmetry. An approximate (isotropic) treatment of cell e.s.d.'s is used for estimating e.s.d.'s involving l.s. planes.
Refinement. Refinement of *F*^2^ against ALL reflections. The weighted *R*-factor *wR* and goodness of fit *S* are based on *F*^2^, conventional *R*-factors *R* are based on *F*, with *F* set to zero for negative *F*^2^. The threshold expression of *F*^2^ > σ(*F*^2^) is used only for calculating *R*-factors(gt) *etc*. and is not relevant to the choice of reflections for refinement. *R*-factors based on *F*^2^ are statistically about twice as large as those based on *F*, and *R*- factors based on ALL data will be even larger.

### Fractional atomic coordinates and isotropic or equivalent isotropic displacement parameters (Å^2^)

**Table:**

	*x*	*y*	*z*	*U*_iso_*/*U*_eq_	
O1	0.92938 (16)	0.39637 (11)	0.93311 (9)	0.0301 (3)	
O2	0.66859 (17)	0.57336 (11)	0.88256 (10)	0.0354 (3)	
O3	1.15083 (16)	0.18178 (12)	0.96436 (10)	0.0330 (3)	
O4	0.49898 (16)	−0.15978 (12)	0.67925 (9)	0.0312 (3)	
O5	0.27400 (16)	−0.18783 (12)	0.84664 (10)	0.0332 (3)	
O6	0.75828 (19)	−0.09713 (14)	0.53681 (10)	0.0403 (3)	
C1	0.7423 (2)	0.44344 (15)	0.89396 (12)	0.0257 (3)	
C2	0.6609 (2)	0.30800 (14)	0.87438 (11)	0.0220 (3)	
H2	0.5548	0.2806	0.9443	0.026*	
C3	0.8484 (2)	0.18248 (15)	0.87829 (12)	0.0235 (3)	
H3	0.8078	0.0875	0.9278	0.028*	
C4	0.9938 (2)	0.24363 (15)	0.93188 (13)	0.0267 (3)	
C5	0.9568 (2)	0.15432 (16)	0.75541 (13)	0.0255 (3)	
H5A	1.0170	0.2446	0.7129	0.031*	
H5B	1.0712	0.0687	0.7623	0.031*	
C6	0.8083 (2)	0.11962 (15)	0.68574 (12)	0.0233 (3)	
H6	0.8836	0.1187	0.6038	0.028*	
C7	0.6239 (2)	0.24430 (14)	0.68056 (11)	0.0217 (3)	
C8	0.5574 (2)	0.33563 (14)	0.76735 (11)	0.0217 (3)	
C9	0.7444 (2)	−0.03861 (15)	0.73173 (12)	0.0243 (3)	
H9	0.8682	−0.1090	0.7553	0.029*	
C10	0.5642 (2)	−0.05583 (16)	0.83299 (12)	0.0263 (3)	
H10A	0.6142	−0.1119	0.9050	0.032*	
H10B	0.4887	0.0441	0.8484	0.032*	
C11	0.6786 (2)	−0.09717 (16)	0.63586 (13)	0.0282 (3)	
C12	0.4273 (2)	−0.14180 (15)	0.79467 (12)	0.0253 (3)	
C13	0.5157 (2)	0.26953 (16)	0.58755 (12)	0.0267 (3)	
H13	0.5581	0.2077	0.5286	0.032*	
C14	0.3478 (2)	0.38295 (16)	0.57969 (13)	0.0298 (3)	
H14	0.2769	0.3981	0.5154	0.036*	
C15	0.2811 (2)	0.47547 (16)	0.66501 (13)	0.0280 (3)	
C16	0.3867 (2)	0.44920 (15)	0.75866 (13)	0.0253 (3)	
H16	0.3418	0.5100	0.8182	0.030*	
C17	0.1005 (3)	0.60085 (19)	0.65557 (16)	0.0397 (4)	
H17A	0.1385	0.6798	0.5899	0.060*	
H17B	0.0630	0.6433	0.7282	0.060*	
H17C	−0.0175	0.5611	0.6425	0.060*	

### Atomic displacement parameters (Å^2^)

**Table:**

	*U*^11^	*U*^22^	*U*^33^	*U*^12^	*U*^13^	*U*^23^
O1	0.0320 (6)	0.0239 (5)	0.0387 (6)	−0.0023 (4)	−0.0146 (4)	−0.0086 (4)
O2	0.0399 (6)	0.0225 (5)	0.0467 (7)	0.0026 (4)	−0.0148 (5)	−0.0122 (5)
O3	0.0293 (6)	0.0322 (6)	0.0406 (6)	−0.0015 (4)	−0.0170 (5)	−0.0054 (5)
O4	0.0339 (6)	0.0336 (6)	0.0306 (5)	−0.0099 (4)	−0.0074 (4)	−0.0095 (4)
O5	0.0295 (6)	0.0309 (6)	0.0399 (6)	−0.0061 (4)	−0.0068 (5)	−0.0052 (5)
O6	0.0437 (7)	0.0467 (7)	0.0346 (6)	−0.0114 (5)	0.0020 (5)	−0.0212 (5)
C1	0.0276 (7)	0.0240 (7)	0.0264 (7)	−0.0009 (5)	−0.0065 (5)	−0.0071 (5)
C2	0.0227 (6)	0.0206 (6)	0.0224 (6)	−0.0017 (5)	−0.0039 (5)	−0.0043 (5)
C3	0.0240 (7)	0.0196 (6)	0.0284 (7)	−0.0017 (5)	−0.0090 (5)	−0.0042 (5)
C4	0.0291 (7)	0.0235 (7)	0.0289 (7)	−0.0032 (5)	−0.0088 (6)	−0.0047 (5)
C5	0.0201 (6)	0.0248 (7)	0.0333 (7)	−0.0018 (5)	−0.0050 (5)	−0.0099 (6)
C6	0.0214 (6)	0.0232 (7)	0.0255 (6)	−0.0010 (5)	−0.0032 (5)	−0.0079 (5)
C7	0.0224 (6)	0.0194 (6)	0.0231 (6)	−0.0048 (5)	−0.0028 (5)	−0.0026 (5)
C8	0.0215 (6)	0.0196 (6)	0.0241 (6)	−0.0033 (5)	−0.0049 (5)	−0.0025 (5)
C9	0.0241 (7)	0.0206 (6)	0.0292 (7)	0.0011 (5)	−0.0072 (5)	−0.0085 (5)
C10	0.0310 (7)	0.0231 (7)	0.0265 (7)	−0.0044 (5)	−0.0087 (6)	−0.0044 (5)
C11	0.0280 (7)	0.0246 (7)	0.0331 (7)	−0.0012 (5)	−0.0050 (6)	−0.0105 (6)
C12	0.0265 (7)	0.0202 (6)	0.0290 (7)	0.0003 (5)	−0.0094 (5)	−0.0031 (5)
C13	0.0308 (7)	0.0253 (7)	0.0254 (7)	−0.0057 (6)	−0.0067 (6)	−0.0036 (5)
C14	0.0323 (8)	0.0283 (7)	0.0304 (7)	−0.0054 (6)	−0.0141 (6)	0.0012 (6)
C15	0.0247 (7)	0.0223 (7)	0.0360 (8)	−0.0018 (5)	−0.0088 (6)	−0.0001 (6)
C16	0.0247 (7)	0.0203 (6)	0.0307 (7)	−0.0018 (5)	−0.0050 (5)	−0.0050 (5)
C17	0.0317 (8)	0.0343 (8)	0.0505 (10)	0.0058 (7)	−0.0165 (7)	−0.0013 (7)

### Geometric parameters (Å, º)

**Table:**

O1—C1	1.3851 (17)	C6—H6	1.0000
O1—C4	1.3865 (17)	C7—C13	1.3956 (19)
O2—C1	1.1915 (17)	C7—C8	1.4027 (18)
O3—C4	1.1935 (17)	C8—C16	1.3970 (18)
O4—C12	1.3850 (17)	C9—C11	1.517 (2)
O4—C11	1.3905 (18)	C9—C10	1.526 (2)
O5—C12	1.1900 (18)	C9—H9	1.0000
O6—C11	1.1862 (18)	C10—C12	1.4991 (19)
C1—C2	1.5207 (19)	C10—H10A	0.9900
C2—C8	1.5200 (18)	C10—H10B	0.9900
C2—C3	1.5344 (18)	C13—C14	1.383 (2)
C2—H2	1.0000	C13—H13	0.9500
C3—C4	1.5031 (19)	C14—C15	1.397 (2)
C3—C5	1.5362 (19)	C14—H14	0.9500
C3—H3	1.0000	C15—C16	1.390 (2)
C5—C6	1.5244 (19)	C15—C17	1.507 (2)
C5—H5A	0.9900	C16—H16	0.9500
C5—H5B	0.9900	C17—H17A	0.9800
C6—C7	1.5165 (18)	C17—H17B	0.9800
C6—C9	1.5533 (19)	C17—H17C	0.9800
			
C1—O1—C4	110.69 (11)	C7—C8—C2	121.09 (12)
C12—O4—C11	110.64 (11)	C11—C9—C10	103.25 (11)
O2—C1—O1	120.19 (13)	C11—C9—C6	110.63 (12)
O2—C1—C2	130.17 (13)	C10—C9—C6	118.92 (11)
O1—C1—C2	109.62 (11)	C11—C9—H9	107.9
C8—C2—C1	114.64 (11)	C10—C9—H9	107.9
C8—C2—C3	116.61 (11)	C6—C9—H9	107.9
C1—C2—C3	103.25 (11)	C12—C10—C9	105.44 (11)
C8—C2—H2	107.3	C12—C10—H10A	110.7
C1—C2—H2	107.3	C9—C10—H10A	110.7
C3—C2—H2	107.3	C12—C10—H10B	110.7
C4—C3—C2	103.97 (11)	C9—C10—H10B	110.7
C4—C3—C5	108.12 (11)	H10A—C10—H10B	108.8
C2—C3—C5	112.08 (11)	O6—C11—O4	120.17 (13)
C4—C3—H3	110.8	O6—C11—C9	129.62 (14)
C2—C3—H3	110.8	O4—C11—C9	110.19 (12)
C5—C3—H3	110.8	O5—C12—O4	120.36 (13)
O3—C4—O1	120.54 (13)	O5—C12—C10	129.79 (13)
O3—C4—C3	129.34 (13)	O4—C12—C10	109.80 (12)
O1—C4—C3	109.98 (11)	C14—C13—C7	121.11 (13)
C6—C5—C3	111.80 (11)	C14—C13—H13	119.4
C6—C5—H5A	109.3	C7—C13—H13	119.4
C3—C5—H5A	109.3	C13—C14—C15	120.87 (13)
C6—C5—H5B	109.3	C13—C14—H14	119.6
C3—C5—H5B	109.3	C15—C14—H14	119.6
H5A—C5—H5B	107.9	C16—C15—C14	118.15 (13)
C7—C6—C5	110.47 (11)	C16—C15—C17	120.88 (14)
C7—C6—C9	112.62 (11)	C14—C15—C17	120.97 (14)
C5—C6—C9	112.35 (11)	C15—C16—C8	121.59 (13)
C7—C6—H6	107.0	C15—C16—H16	119.2
C5—C6—H6	107.0	C8—C16—H16	119.2
C9—C6—H6	107.0	C15—C17—H17A	109.5
C13—C7—C8	118.59 (12)	C15—C17—H17B	109.5
C13—C7—C6	119.62 (12)	H17A—C17—H17B	109.5
C8—C7—C6	121.79 (12)	C15—C17—H17C	109.5
C16—C8—C7	119.69 (13)	H17A—C17—H17C	109.5
C16—C8—C2	119.14 (12)	H17B—C17—H17C	109.5
			
C4—O1—C1—O2	−175.65 (13)	C3—C2—C8—C16	−179.58 (12)
C4—O1—C1—C2	5.59 (15)	C1—C2—C8—C7	124.46 (13)
O2—C1—C2—C8	40.1 (2)	C3—C2—C8—C7	3.66 (18)
O1—C1—C2—C8	−141.25 (12)	C7—C6—C9—C11	78.97 (14)
O2—C1—C2—C3	168.05 (15)	C5—C6—C9—C11	−155.52 (11)
O1—C1—C2—C3	−13.35 (14)	C7—C6—C9—C10	−40.22 (17)
C8—C2—C3—C4	141.97 (12)	C5—C6—C9—C10	85.29 (15)
C1—C2—C3—C4	15.30 (13)	C11—C9—C10—C12	8.01 (14)
C8—C2—C3—C5	25.43 (16)	C6—C9—C10—C12	130.94 (12)
C1—C2—C3—C5	−101.23 (12)	C12—O4—C11—O6	−177.29 (13)
C1—O1—C4—O3	−178.89 (13)	C12—O4—C11—C9	3.61 (15)
C1—O1—C4—C3	5.03 (15)	C10—C9—C11—O6	173.67 (15)
C2—C3—C4—O3	171.22 (15)	C6—C9—C11—O6	45.4 (2)
C5—C3—C4—O3	−69.50 (19)	C10—C9—C11—O4	−7.34 (15)
C2—C3—C4—O1	−13.15 (15)	C6—C9—C11—O4	−135.61 (11)
C5—C3—C4—O1	106.13 (13)	C11—O4—C12—O5	179.67 (12)
C4—C3—C5—C6	−169.56 (11)	C11—O4—C12—C10	1.92 (15)
C2—C3—C5—C6	−55.55 (15)	C9—C10—C12—O5	176.04 (14)
C3—C5—C6—C7	55.19 (15)	C9—C10—C12—O4	−6.49 (15)
C3—C5—C6—C9	−71.49 (14)	C8—C7—C13—C14	0.7 (2)
C5—C6—C7—C13	154.16 (12)	C6—C7—C13—C14	−179.52 (13)
C9—C6—C7—C13	−79.31 (15)	C7—C13—C14—C15	−0.2 (2)
C5—C6—C7—C8	−26.09 (17)	C13—C14—C15—C16	−0.7 (2)
C9—C6—C7—C8	100.44 (15)	C13—C14—C15—C17	178.93 (14)
C13—C7—C8—C16	−0.3 (2)	C14—C15—C16—C8	1.1 (2)
C6—C7—C8—C16	179.93 (12)	C17—C15—C16—C8	−178.53 (13)
C13—C7—C8—C2	176.43 (12)	C7—C8—C16—C15	−0.6 (2)
C6—C7—C8—C2	−3.3 (2)	C2—C8—C16—C15	−177.42 (12)
C1—C2—C8—C16	−58.78 (16)		
